# How Free Swimming Fosters the Locomotion of a Purely Oscillating Fish-like Body

**DOI:** 10.3390/biomimetics8050401

**Published:** 2023-09-01

**Authors:** Damiano Paniccia, Luca Padovani, Giorgio Graziani, Claudio Lugni, Renzo Piva

**Affiliations:** 1 Department of Mechanical and Aerospace Engineering, Sapienza University, 00184 Rome, Italy; damiano.paniccia@uniroma1.it (D.P.); luca.padovani@uniroma1.it (L.P.); renzo.piva@uniroma1.it (R.P.); 2 Leonardo S.p.A., Piazza Monte Grappa 4, 00195 Rome, Italy; 3 CNR-INM, Marine Technology Research Institute, 00128 Rome, Italy; claudio.lugni@cnr.it; 4 Marine Technology Department, NTNU, NO-7491 Trondheim, Norway

**Keywords:** aquatic locomotion, swimming performance, fish tail damage, recoil, fluid–structure interactions, biomimetic fluid dynamics

## Abstract

The recoil motions in free swimming, given by lateral and angular rigid motions due to the interaction with the surrounding water, are of great importance for a correct evaluation of both the forward locomotion speed and efficiency of a fish-like body. Their contribution is essential for calculating the actual movements of the body rear end whose prominent influence on the generation of the proper body deformation was established a long time ago. In particular, the recoil motions are found here to promote a dramatic improvement of the performance when damaged fishes, namely for a partial functionality of the tail or even for its complete loss, are considered. In fact, the body deformation, which turns out to become oscillating and symmetric in the extreme case, is shown to recover in the water frame a kind of undulation leading to a certain locomotion speed though at the expense of a large energy consumption. There has been a deep interest in the subject since the infancy of swimming studies, and a revival has recently arisen for biomimetic applications to robotic fish-like bodies. We intend here to apply a theoretical impulse model to the oscillating fish in free swimming as a suitable test case to strengthen our belief in the beneficial effects of the recoil motions. At the same time, we intend to exploit the linearity of the model to detect from the numerical simulations the intrinsic physical reasons related to added mass and vorticity release behind the experimental observations.

## 1. Introduction

In the initial decades of the past century, starting from some experimental observations, several authors, such as Breder [1] and Gray [2,3,4], proposed a very brilliant analysis of the wave-like nature of fish swimming that was deepened later on with theoretical models looking for the best thrust production and the most efficient swimming performance. The caudal fin was recognized as the essential tool for the generation of the desired body deformation and its important function was described to obtain, throughout muscular contraction and energy transmission to the fluid, a gentle and efficient self-propulsion. For a better comprehension of this subtle issue, several conditions of a reduced action of the tail were instrumentally analyzed to show how a lower swimming performance was provided. More recently, many authors analyzed, for the survival of damaged fish, a partial functionality of the tail up to its complete loss [5,6,7,8,9,10,11]. In all these cases, the rear end of the body did not achieve the proper deformation by favoring instead a kind of oscillating motion that is purely symmetric in the body frame and presumably unable to efficiently generate a forward thrust. However, a comparable locomotion, even though more energy consuming, was observed in the experimental investigations for fish swimming in a water channel. From a theoretical point of view, this observation is perfectly consistent with the presence of recoil motions, i.e., further rigid motions of the body beyond the main forward locomotion, generated by the fluid interaction to satisfy the equilibrium of the entire system as underlined in the early 1960s by Lighthill [12] and Wu [13]. Once in the water frame, these recoil motions are seen to modify the oscillating deformation of the body to establish again a sort of favorable undulation (see also [14]). In other words, by letting a purely oscillating fish-like body experience all the recoil motions, we find a partial recovery of the self-propulsion capability which, on the contrary, is severely reduced if the swimmer is axially constrained.

The aim of the paper is to gain, even with this unfavorable body deformation, a definitive proof of the improved performance of free swimming by meaningful numerical results for a simple physical approximation of the fish-like body in the unbounded water domain. Actually, we plan to give some answers about the unexpected recovery of self-propulsion, with respect to the axially constrained motion, through the maneuvers dictated by free swimming when the fish has the possibility to select the most suitable style for its locomotion. To this purpose, an impulse potential model with concentrated vorticity leads to neat numerical simulations that are able to provide new physical interpretations of the above-mentioned seminal findings. Specifically, by exploiting the linearity of the adopted model, we may separately isolate the effect of the added mass and of the vorticity release to evaluate their separate contribution to the improved performance. The significance of the recoil, as a beneficial effect on the performance, was previously analyzed ([15,16]) in the realm of plain undulatory swimming by considering several constrained motions, with one or more velocity components totally prevented to force the fish along a compatible trajectory. Analogously, the beneficial effect of the recoil motion, once combined with the prescribed body deformation, has been analyzed for the flapping tail propeller in a typical oscillatory swimming [14]. The comprehension of all the above issues may be of a certain interest for the design of biomimetic swimmers to suggest the geometrical configuration and the kinematical parameters to obtain the most appropriate swimming style for the desired application.

## 2. Materials and Methods

### 2.1. Mathematical Model

The self-propulsion of a free swimming body B is modeled by considering a two-dimensional flow in an unbounded fluid domain $V_{\infty }$. Only internal actions are exchanged between the swimming body and the surrounding fluid, which is otherwise quiescent. We adopt the impulse formulation (see, e.g., [17,18,19]) for both linear and angular fluid momenta which are expressed in terms of potential and vortical contributions. The mathematical model, fully described in [20], is shortly summarized below for a better understanding of the described results.

We consider an impermeable, flexible body whose bounding surface ∂B is moving with a fully prescribed velocity $u_{b}$; hence, we restrict our study only to a one-way coupling of the fluid–structure interaction. Concerning the fluid, we assume an unbounded 2D incompressible flow field, with density ρ, whose velocity vanishes at the far field boundary. The force $F_{b}$ and the moment $M_{b}$ acting on the body are expressed through the time derivatives of the total linear impulse, p, and angular impulse, π:

(1) $$F_{b}=-\frac{dp}{dt}\;\;\;\;\;\;\;\;\;M_{b}=-\frac{d\pi }{dt}$$

where p and π are defined, by using the well-known vector identities for an unbounded two-dimensional fluid volume, as

(2a) $$\begin{array}{cc} p & =\int_{V_{\infty }}\rho x\times \omega dV+\int_{\partial B}\rho x\times \left(n\times u^{+}\right)dS \end{array}$$

(2b) $$\begin{array}{cc} \pi & =-\frac{1}{2}\left[\int_{V_{\infty }}\rho \;{\left|x\right|}^{2}\omega \;dV+\int_{\partial B}\rho \;{\left|x\right|}^{2}\left(n\times u^{+}\right)\;dS\right] \end{array}$$

In these expressions, the integrals over the external boundary receding to infinity have been proven to vanish, ω is the vorticity and $u^{+}$ stays for the limiting value of the fluid velocity on ∂B. The normal to ∂B, n, points into the fluid domain $V_{\infty }$, which extends to infinity.

By introducing the scalar potential ϕ and the (solenoidal) vector potential Ψ, the velocity field is expressed as the sum of the acyclic (∇ϕ) and vorticity-related ($u_{w}$) components through a Helmholtz decomposition:

(3) $$u=\nabla \phi +\nabla \times \Psi =\nabla \phi +u_{w}$$

ϕ and Ψ are easily solved by imposing the impermeable boundary condition on ∂B and vanishing velocity at infinity. We may then express p in terms of its potential and vortical contributions as $p=p_{\phi }+p_{v}$ where the potential impulse $p_{\phi }$ is given by

(4) $$p_{\phi }=-\rho \;\int_{\partial B}\phi \;n\;dS$$

and the vortical impulse is

(5) $$p_{v}=\int_{V_{\infty }}\rho \;x\times \omega dV\;+\int_{\partial B}\rho \;x\times \left(n\times u_{w}\right)\;\mathrm{dS}$$

Similarly, by using appropriate vector identities, the angular impulse can be split into the potential and vortical parts as $\pi =\pi_{\phi }+\pi_{v}$ where the former is given by

(6) $$\pi_{\phi }=-\frac{1}{2}\int_{\partial B}\rho \;{\left|x\right|}^{2}\left(n\times \nabla \phi \right)\;dS=-\rho \;\int_{\partial B}x\times \phi n\;dS$$

and the angular vortical impulse is

(7) $$\pi_{v}=-\frac{1}{2}\int_{V_{\infty }}\rho \;{\left|x\right|}^{2}\omega \;dV-\frac{1}{2}\int_{\partial B}\rho \;{\left|x\right|}^{2}\left(n\times u_{w}\right)\;dS$$

To evaluate the planar motion of the body, we start from the conservation of the linear and angular momenta and, by assuming null initial conditions, we have

(8) $$\int_{B}\rho_{b}\;u_{b}\;dV+\int_{V}\rho \;u\;dV=0$$

(9) $$\int_{B}\rho_{b}\;x\times u_{b}\;dV+\int_{V}\rho \;x\times u\;dV=0$$

The total motion of the body is given by the sum of the prescribed deformation (shape variation with velocity $u_{\mathrm{sh}}$) plus the motion of the frame with the origin in the center-of-mass (translational, $u_{\mathrm{cm}}$, and rotational, Ω, velocity)

(10) $$u_{b}=u_{sh}+u_{cm}+\Omega \times x^{\prime }$$

where $x^{\prime }$ is the position vector in the body frame, i.e., $x=x_{\mathrm{cm}}+x^{\prime }$.

The prescribed body deformation has to conserve linear and angular momenta even in the absence of the surrounding fluid so that the following two conditions have to be satisfied to ensure that no rigid body motion may be achieved by an isolated body.

(11) $$\int_{B}\rho_{b}u_{sh}\;dV=0$$

(12) $$\int_{B}\rho_{b}x^{\prime }\times u_{sh}\;dV=0$$

In case Equations (11) and (12) are not satisfied, the resulting spurious motions of the center-of-mass, known as geometrical recoil motions, may be removed as detailed in [14]. By considering that the second term in Equation (8) is the force acting on the fluid, which is opposite to the force on the body, and by combining with Equation (2a), we obtain:

(13) $$m_{b}\;u_{cm}+\;p=0$$

and, similarly, from Equations (9) and (2b):

(14) $$I_{zz}\;\Omega +\pi^{\prime }=0$$

where $m_{b}$ is the body mass and the angular impulse is recast from Equation (2b) in terms of the distance $x^{\prime }$ as $\pi^{\prime }=\left(\pi -x_{o}\times p\right)\cdot e_{3}$

The scalar potential introduced by the Helmholtz decomposition is furtherly split as $\phi =\phi_{sh}+\phi_{loc}$, (see [21,22,23]) where $\phi_{sh}$ is given by the imposed deformation velocity $u_{sh}$ and $\phi_{loc}$ follows from the combination of the locomotion linear and angular velocities, according to the related boundary conditions on $S_{b}$

$$\frac{\partial \phi_{sh}}{\partial n}=u_{sh}\cdot n\;\;\;\;\;\;\;\frac{\partial \phi_{loc}}{\partial n}=\left(u_{cm}+\Omega \times x^{\prime }\right)\cdot n$$

Analogously, the linear and angular impulses are given by:

(15) $$p_{\phi }=p_{sh}+p_{loc}\;\;\;\;\;\;\;\pi_{\phi }^{\prime }=\pi_{sh}^{\prime }+\pi_{loc}^{\prime }$$

Finally, the locomotion impulses, $p_{loc}$ and $\pi_{loc}^{\prime }$, may be expressed in terms of the added mass coefficients reported in the classical treatises (see, e.g., [24]). For a body motion with linear velocity $u_{cm}$ and angular velocity Ω, we consider the Kirchhoff base potentials $\Phi_{j}$ defined through the boundary conditions

(16) $$\frac{\partial \Phi_{1}}{\partial n}=n\cdot e_{1}\;\;\;\;\;\;\;\frac{\partial \Phi_{2}}{\partial n}=n\cdot e_{2}\;\;\;\;\;\;\;\frac{\partial \Phi_{3}}{\partial n}=x^{\prime }\times n\cdot e_{3}$$

to have $\phi_{loc}={\dot{x}}_{0}\Phi_{1}+{\dot{y}}_{0}\Phi_{2}+\Omega \Phi_{3}$. It follows for the added mass coefficient $m_{ij}$ the expression

(17) $$m_{ij}=\rho \int_{S_{b}}\Phi_{i}\;\frac{\partial \Phi_{j}}{\partial n}\;dS$$

Once we recast the locomotion equations in a reference frame fixed to the body, and we use capital letters for the unknowns linear ($V_{1},V_{2}$) and angular (Ω) velocities in this frame, we may bring to the right-hand side (hereafter r. h. s.) the known terms due to body shape deformation (${P_{sh}}_{1},{P_{sh}}_{2},\Pi_{sh}$) and released vorticity (${P_{v}}_{1},{P_{v}}_{2},\Pi_{v}$). So, the body motion is obtained by solving at each time step the system

(18) $$\left\{\begin{array}{cc} V_{1}\;\left(m_{11}+m_{b}\right)+V_{2}\;m_{12}+\Omega \;m_{13} & =-{P_{sh}}_{1}-{P_{v}}_{1}\\ V_{1}\;m_{21}+V_{2}\;\left(m_{22}+m_{b}\right)+\Omega \;m_{23} & =-{P_{sh}}_{2}-{P_{v}}_{2}\\ V_{1}\;m_{31}+V_{2}\;m_{32}+\Omega \left(m_{33}+I_{zz}\right) & =-\Pi_{sh}-\Pi_{v} \end{array}\right.$$

**Figure 1:**
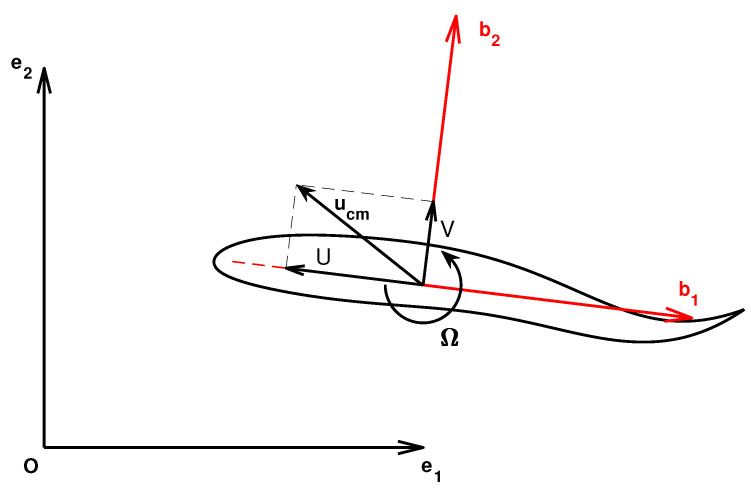
Ground (black) and body (red) reference frames, linear and angular locomotion velocities and center-of-mass velocity.

To be consistent with their classical denomination, the velocity components are renamed as $U=-V_{1}$ and $V=V_{2}$. A sketch with some relevant quantities is reported in Figure 1 where the ground-fixed frame $\left\{e_{1},e_{2}\right\}$ as well as the body frame $\left\{b_{1},b_{2}\right\}$ are shown. The origin of the body frame is in the center-of-mass. In the same figure, the locomotion velocities, linear *U*, *V* and angular Ω are represented.

### 2.2. Numerical Method and Fish Kinematics

A simple 2D inviscid model with concentrated vorticity has been used to achieve neat and simple results. Specifically, the solutions are obtained by an unsteady potential code based on the Hess and Smith approach [25], while the wake release is taken into account by following the procedure described in [26]. The approach consists of approximating the body surface by a finite number of panels, each one with a local, uniformly distributed, source strength and all with a global, uniform vorticity strength. For *n* panels, there are *n* unknown source distributions and one unknown vorticity distribution, while the impermeability condition on each panel and the Kutta condition form a set of n+1 equations for the n+1 unknowns. According to Kelvin’s theorem, any change in circulation about the body results in the release of vorticity into the wake through a wake panel attached to the trailing edge. At the end of each time step, this wake panel is lumped into a point vortex which is shed into the wake with the flow velocity. The time history of the contributions to the forward velocity is reported in Figure A3. The non-circulatory terms due to the shape deformation, both linear and angular, reach an immediate steady-state average value, which is quantitatively not very important but essential to trigger the entire process, leading to the vorticity release. On the other end, the terms due the released vorticity, i.e., ${P_{v}}_{1},{P_{v}}_{2},\Pi_{v}$ appearing on the r. h. s. in Equation (18), keep growing in time to finally give the major contribution to the steady-state locomotion speed.

### 2.3. Prescribed Deformation

To illustrate the movements of the rear end of the body relative to the head for the intact and for the damaged whiting, Gray [4] used several fish photographs by overlapping their heads to highlight the difference due to the presence or to the absence of the tail. For a qualitative reproduction of the experimental results, we selected the body deformations to match the snapshots in his paper. The two-dimensional body is represented by an undulating foil, whose shape at rest corresponds to an NACA0012 airfoil with a total length *L*. As a carangiform swimming fish, the intact whiting deformation is prescribed, in the body-fixed frame, by a well-known analytical expression for the lateral displacement y(x,t) of the midline, which was obtained by fitting data from direct fish observations (see [27]):

(19) $$y\left(x,t\right)=A\left(x\right)\;\sin\left[2\pi (x/\lambda -f\;t)\right]$$

where λ is the wavelength, *f* is the undulation frequency and the factor A(x) is a polynomial amplitude modulation given by

(20) $$A\left(x\right)=a_{0}\left[ax^{2}+bx+c\right]$$

where $a_{0}=0.1$ is the maximum tail-beat amplitude. The coefficients *a*, *b* and *c* are usually selected to obtain the required deformation, and for the carangiform style, the most commonly used values (see, e.g., [28,29]) are: a=0.2,b=−0.825,c=1.625. The coefficients in the kinematics in Equation (20) can also be identified as described in [30].

For the damaged fish deformation, we followed instead the path suggested in [31], once the prescribed motion of the caudal fin is removed, to obtain a pure oscillation up to the rear end of the body whose total length is preserved. In detail, while the initial part of the body (2/3L) is fixed, the remaining flexible part is divided into *N* segments of length $l_{i}$, whose maximum lateral and rotational motions are $A_{i}$ and $\psi_{i}$, respectively. These coefficients have been selected as described in [31] as:

(21) $$\left\{\begin{array}{cc} \; & A_{1}=0\\ \; & A_{i}=A_{r}{\left(\frac{\sum_{j=1}^{i-1}l_{j}}{1/3\;L}\right)}^{2}\;\;\;\;\;i=2,\ldots ,\;N\;\;\;\;\;\; \end{array}\right.\left\{\begin{array}{cc} \; & \psi_{i}=\arcsin\frac{A_{i+1}-A_{i}}{l_{i}}\;\;\;\;\;i=1,\ldots ,\;N-1\\ \; & \psi_{N}=\arcsin\frac{A_{r}-A_{N}}{l_{N}} \end{array}\right.$$

where $A_{r}$ is the maximum amplitude at the body rear end, which is equal to 0.16 in the present work. Finally, the oscillating deformation leading to a constant length of the body during the prescribed motion is given by:

(22) $$\left\{\begin{array}{cc} \Psi_{i}\left(t\right) & =\psi_{i}\;sin\left(2\pi ft\right)\\ x_{i+1}\left(t\right) & =x_{i}+l_{i}\;cos\left[\Psi_{i}\left(t\right)\right]\;\;\;\;\;i=1,\ldots ,\;N\\ y_{i+1}\left(t\right) & =y_{i}+l_{i}\;sin\left[\Psi_{i}\left(t\right)\right] \end{array}\right.$$

Let us remark that prescribing the body deformation requires two additional mathematical conditions given by the conservation of linear and angular momenta to avoid spurious external actions (see [14]).

## 3. Results

**Figure 2:**
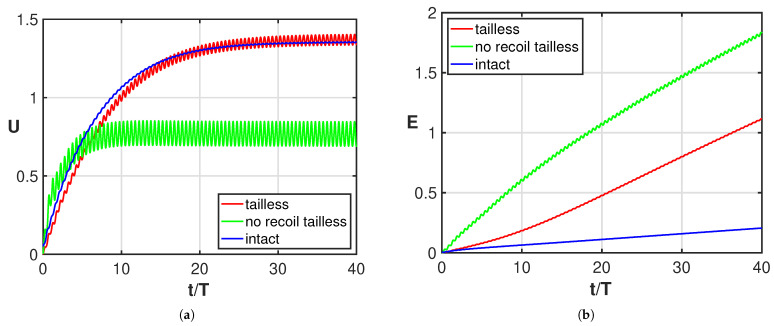
Time behavior of (**a**) forward velocity and (**b**) energy consumption for the intact fish (blue), for the tailless fish (red) and for the constrained tailless fish (green).

The undulating deformation of a fish-like body is expected to give an efficient style of swimming with reduced energy consumption. Although different deformations characterize different species, for the great majority of them, the tail plays an essential role in providing the optimal kind of undulation to the entire body or to just part of it [32,33,34]. Starting from these concepts, several authors, to prove its great importance, considered a tail with a reduced functionality from partial damage to the complete loss [5,6,7,8,9,10,11]. Complete experimental evidence was given by Gray [4] who documented in a large number of photographs the successive positions of a swimming whiting, in its normal configuration, in comparison with the positions taken by the same fish once its caudal fin was lost. The resulting sketch reported in his paper shows how the tailless fish does not undulate anymore, while a symmetric oscillation about its axial direction in the body-fixed frame starts to take place with an amplitude increasing toward the rear end. From the experimental observations of the fish placed in water, he deduced that despite the symmetric oscillation, the main effect is still a comparable forward self-propulsion even though it has a lower efficiency. By accounting for the recoil motions due to fluid–body interactions within the above illustrated two-dimensional model, we provide here a straightforward explanation of the behavior of the oscillating fish kinematics as originally proposed by Gray. In fact, starting from the prescribed deformations for both the undulating and the oscillating whiting, we may directly obtain very accurate evaluations of the displacement in the inertial frame with our simple approximation. As a first step, we show in Figure 2a the asymptotic swimming velocity achieved by the oscillating fish, which is quite similar to the one reached by the undulating fish, while the energy consumption is clearly much larger as shown in Figure 2b. In the same figure, we report the solution for the tailless fish obtained by preventing both the lateral and angular recoil motions, i.e., with the center-of-mass allowed to move only along the forward direction. In Figure 2a, we may notice that for the tailless fish, a lower locomotion velocity is reached for the constrained motion (green) with respect to the free swimming mode (red). As shown in Figure 2b, the energy consumption is significantly larger for both tailless fish motions with a larger value for the constrained gait.

**Figure 3:**
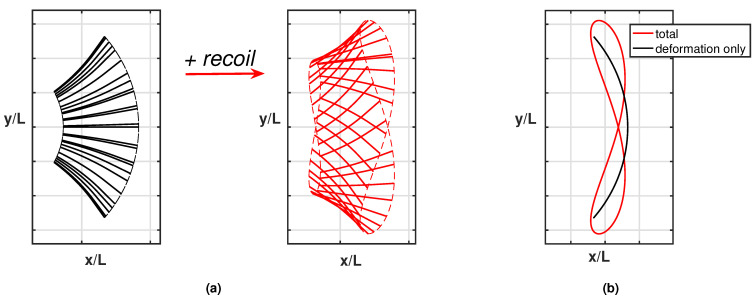
(**a**) Ensemble of the rear-end configurations during the oscillation cycle for the tailless fish in the body-fixed frame (black) and in the inertial frame (red), i.e., accounting for the recoil motions. For completeness, see also the animation (Supplementary Material Movie S1). (**b**) Envelopes of the trailing edge positions along an oscillation cycle in the locomotion frame of reference for the tailless fish: prescribed deformation only (black) and total motion accounting for recoil (red).

Whilst the current results reproduce pretty well the experimentally observed phenomena, a further step is helpful to understand how the detailed aspects of the fish kinematics are changing from one case to the other. As is well known, the motion of the caudal fin may be represented by a flapping foil whose heave and pitch are assigned with a proper phase lag to achieve a satisfactory efficiency [35,36]. When the caudal fin is partially or completely inactive, the posterior end of the fish assumes a vicarious function, which is still oscillating but with no phase lag between the heave and pitch components in the body-fixed frame. However, in the water frame, the lateral and angular recoil motions reintroduce a sort of phase lag which is able to recover a sufficient locomotion performance otherwise severely reduced [16]. The above-described conditions are illustrated in Figure 3a for the tailless fish, where the envelope of the body rear-end oscillation is represented both in the body-fixed and in the water frame.

We may appreciate from these figures how, in the presence of recoil, the rear end of the tailless fish achieves a phase lag between heave and pitch components to move in a way similar to the tail of the undulating fish. In other words, the presence of recoil motions allows the oscillating fish to recover a certain locomotion capability since its kinematics is modified to approximate the one for undulatory motion. The animation for the full cycle (Supplementary Material Movie S1) shows in a simple way the phase lag between heave and pitch by following the time evolution of the flapping segments. The resultant figure-of-eight formed by the trailing edge also confirms the wave-like behavior which has been obtained. Actually, any point of the midline of an inextensible body performing an undulatory deformation moves on a path forming a figure-of-eight in the locomotion frame of reference, i.e., in a body-fixed frame translating with the forward velocity.

We report in the Appendix, for the sake of clarity, a reasoning previously introduced [14] for a flapping foil, which shows how the combination of heave and pitch with a π/2 phase lag may be associated, under certain conditions, to an undulatory motion with a well-defined phase velocity.

**Figure 4:**
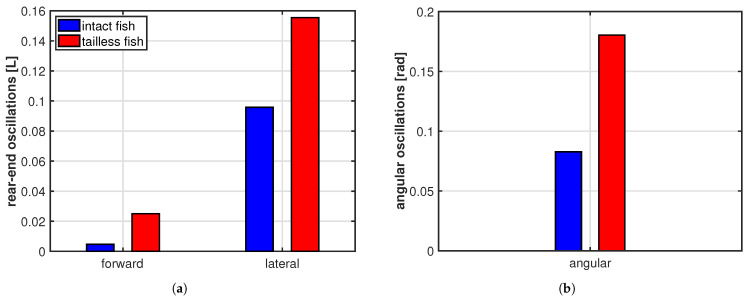
(**a**) Rear-end oscillation amplitude along the forward and the lateral direction; (**b**) whole body angular oscillation amplitude about the center of mass.

As clearly illustrated in Figure 3b, the trailing edge of the oscillating fish does not describe this peculiar path unless the whole motion, including the recoil, is considered. For completeness, we notice that the trailing edge paths for the undulating fish show a figure-of-eight in both frames of reference according to the undulatory character of the deformation. From the comparison of the oscillation amplitudes reported in Figure 4 for the two cases, we may see undoubtedly larger values for the oscillating body, consistently with its need to perform more drastic maneuvers, leading to a larger energy consumption, to gain a comparable locomotion speed. Recently, experimental biologists have highlighted the oscillations of the center-of-mass as a tool to characterize different styles of swimming and the related performances [37,38].

It is interesting to understand from the mathematical model how such drastic maneuvers are able to foster the forward velocity and hence the fish locomotion. To this purpose, we consider the first equation of the system (18) and we move to the r. h. s. the two terms given by the coupling of the lateral and angular motions with the related added mass coefficients. Beyond the predominant role of the vortical impulse, we may appreciate, as largely discussed in a previous paper about the C-start maneuver [14], that the term $\Omega \;m_{13}$ is going to assume an important role which allows for a significant momentum transfer to the forward direction, thus incrementing the resulting locomotion. On the other side, the potential impulse given by the prescribed deformation, due to its symmetrical behavior, leads to a null average value, even though its role is essential to trigger the entire process leading to the vorticity release. The numerical results showing the values of the terms on the r. h. s.of the equation are reported in Figure A3 of the Appendix A to assess their quantitative contributions to the generation of the steady-state locomotion speed. We like to underline here again that the above physical reasoning is provided by the linearity of the present model, which gives the possibility to separate the added mass and the vortical contributions to understand their influence along the fish maneuvers.

**Figure 5:**
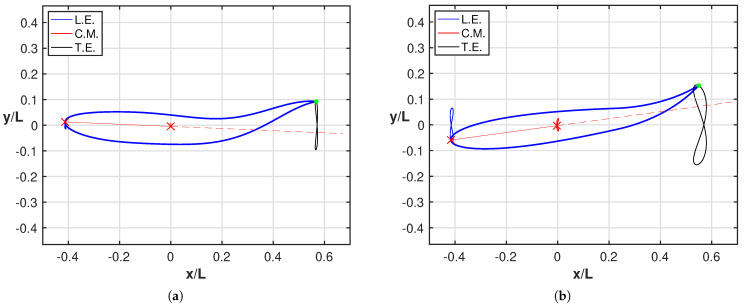
Figures-of-eight trajectories of the leading edge (blue), of the center-of-mass (red) and of the trailing edge (black) in the locomotion frame for (**a**) the intact and (**b**) the tailless fish. The reported snapshots illustrate the maximum inclination of the fish anterior part represented by the straight line (red) connecting the leading edge with the center-of-mass. See the tailless fish animation and the intact fish animation (Supplementary Material Movie S2 and Movie S3).

As a further point, it is worth to compare for both cases the angle of attack α at the trailing edge, whose relevance has been largely recognized in the literature to show how smaller values of α leads to lower values of the cost of transport [39,40]. For further details, the two figures reported in Figure A2 in Appendix A show a much larger angle of attack for the oscillating fish consistently with the larger energy consumption reported in Figure 2b. An analogous behavior for the angle of attack at the head confirms the need to perform very large maneuvers to reach a satisfactory locomotion. This behavior is even more evident in Figure 5 where we report all together the figures-of-eight traced by the leading edge (L.E.), center-of-mass (C.M.) and trailing edge for both fishes together with their related animations to understand how the style of swimming was influenced by the maneuvers. The leading edge and the center-of-mass are connected by a straight line, whose extension is represented by the dashed line, to give a first sight evaluation of the maximum inclination of the fish anterior part. A few phenomena clearly appear from these figures: first, as anticipated, we notice a much larger amplitude of the three figures-of-eight for the oscillating fish. Second, the rotation of the anterior part is notably more pronounced in Figure 5b with respect to Figure 5a. Third, from the animations, it can be appreciated that for the undulating fish, the tail and the head positions are in phase, while the opposite occurs for the oscillating fish to give rise to the large maneuvers mentioned before, which implies a large energy consumption.

**Figure A1:**
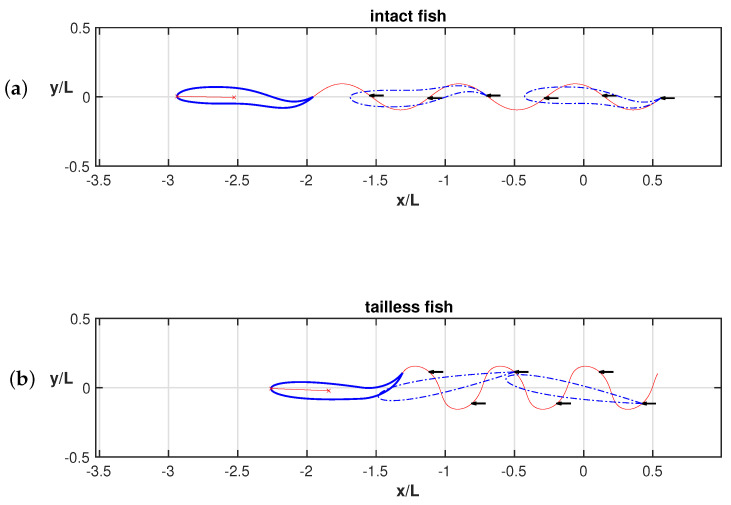
Rear end trajectories for the intact fish (**a**) and for the tailless fish (**b**) with the small arrows located where the alignment condition is verified and some corresponding body configurations are shown.

**Figure A2:**
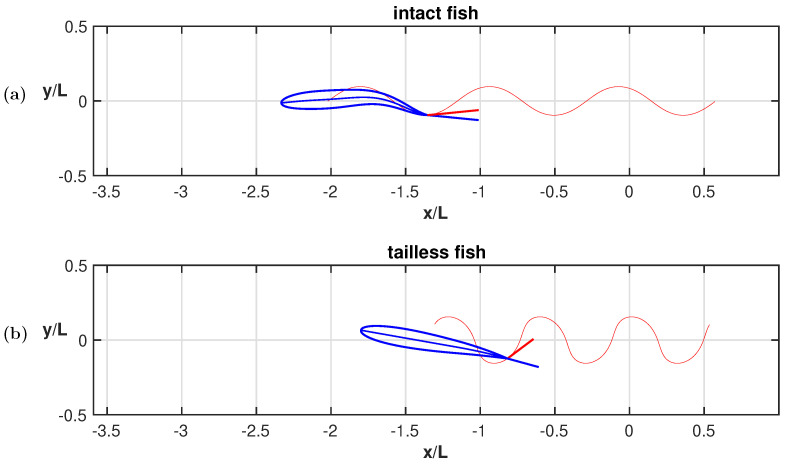
Maximum angle of attack of the rear end for (**a**) the tailless fish and (**b**) the intact fish together with the trace (red) of the trailing edge trajectory.

**Figure A3:**
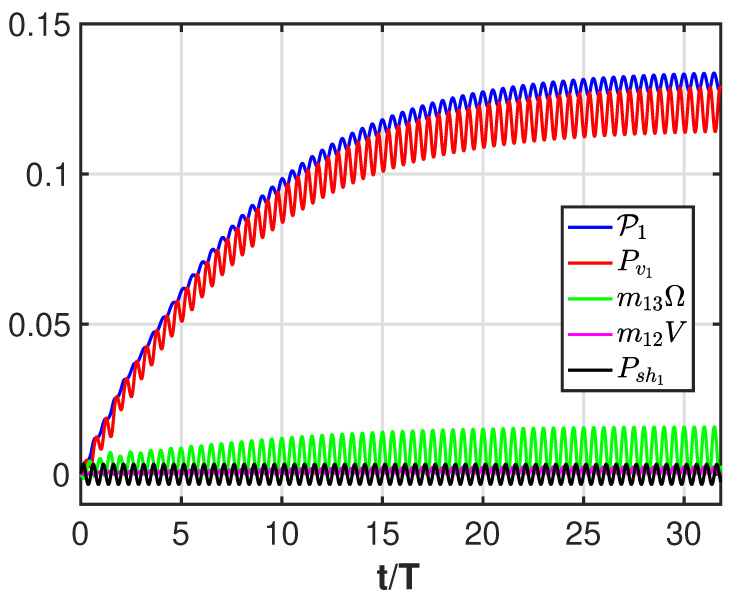
Time history of the contributions to the forward velocity referred to in (A6): total contribution (blue), vortical contribution (red), angular velocity added mass (green), lateral velocity added mass (purple), shape deformation (black).

As a natural consequence, the alignment of the oscillating fishlike body, obtained when the rear end is crossing the dashed straight line, occurs in positions quite displaced from the forward axis, as it can be easily visualized by looking at the trailing edge trajectory. An interesting representation, inspired by the experimental observations [4] and instrumental for clarifying this issue, is reported in Figure A1 of the Appendix A.

**Figure 6:**
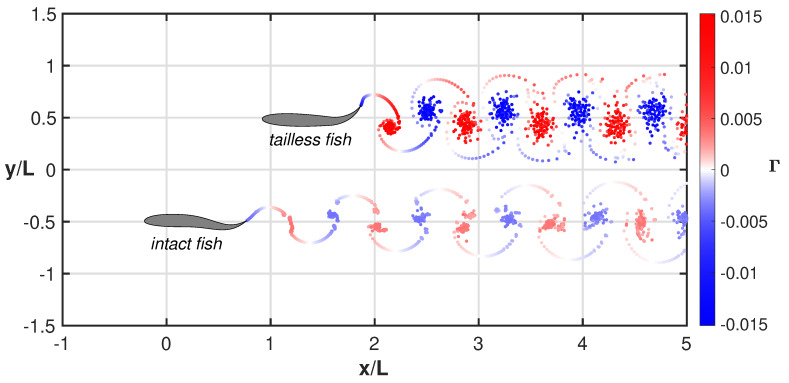
Comparison between the wake structures for the tailless and the intact fish. The elapsed time is the same for both motions. See the complete animation (Supplementary Material Movie S4).

For a quick insight, the swimming styles of the two fishes together with the released vortical wakes are represented in comparison in Figure 6 and related animation (see Supplementary Material Movie S4) where a sort of swimming race clearly illustrates, in a synthetic way, their overall performance. The significant difference between the two vortical wakes gives an immediate feeling of the energy consumption of the two gaits even though the footprints corresponding to the vortex agglomertion are not clearly representing the complex phenomena along the swimming maneuvers [41,42,43].

## 4. Discussion

The oscillating fish has been taken as a significant test case to prove the relevance of the recoil motions and their influence on the style and the performance of fish swimming. We did consider in a previous work [16] the numerical simulations for undulatory free swimming in comparison with constrained gaits obtained by preventing partially or completely the recoil motions. An analogous study [14] was conducted to ascertain the influence of recoil motion once combined with the prescribed kinematics of a flapping foil. In both cases, the main findings suggested that any suppression of these motions generated by the interaction with the surrounding fluid has an adverse influence on the performance, which is in contrast with the unfavorable effect of the recoil frequently mentioned in the literature [44,45,46,47,48]. In the present paper, we show how the recoil is beneficial even for very unusual conditions, like the pure oscillating fish, which occurs for a severe damage of the tail, as indicated in several seminal papers on the subject. The mismatch about the role of the recoil is probably due to its definition as a unique rigid body displacement given by the fluid recoil, due to the interaction with the fluid, and by the geometrical recoil, which is required to satisfy the equilibrium in Equations (11) and (12) for the prescribed deformation [49]. Once the latter are *a priori* satisfied, as in the present model, the remaining fluid recoil has a positive influence on the swimming performance, as confirmed here for the locomotion of the oscillating fish. The results in Figure 3 and related animation have, in our opinion, a central importance since they show how the recoil modifies the rear-end oscillation to recover a phase difference essential for inducing an undulation able to generate a fair self-propulsion. The animations related to Figure 5 give an immediate perception of the different style of swimming adopted by the oscillating fish, which is characterized by very drastic maneuvers, which are strictly required to reach a significant locomotion. The competition between two fishes with undulating and oscillating deformations, respectively, is shown in Figure 6 (and related animation) to release a much stronger vortical wake for the latter which leads to a larger expended energy. In fact, the comparison of the vortical wakes gives an immediate idea of the drag experienced by the two bodies since a larger intensity of the released vorticity structures directly leads to larger energy consumption in terms of excess energy [20,22]. As a final comment, we are confident to have definitely proven the absolute relevance of the recoil motions for a correct evaluation of the swimming performance after showing how the behavior of the purely oscillating fish is recovered when the free swimming mode is used.

## Data Availability

Not applicable.

## Supplementary Materials

The following supporting information can be downloaded at: https://www.mdpi.com/article/10.3390/biomimetics8050401/s1, Supplementary Material Movie S1, Supplementary Material Movie S2, Supplementary Material Movie S3, Supplementary Material Movie S4.

## Appendix A

### Appendix A.1. Flapping–Undulation Analogy

In a previous paper [14], we have shown that the flapping motion given by the combination of a heaving plus a pitching motion of each body segment could be interpreted as an undulating motion provided that heave lags pitch by a phase angle π/2. Upon considering the caudal fin only, we may prescribe its flapping motion through the combination of a heaving motion of the peduncle $y_{p}\left(t\right)$ and a pitching motion about the peduncle itself given by θ(t). The pitch motion θ(t) has a phase angle ϕ=π/2 with respect to the heave motion, so to have

(A1) $$\begin{array}{cc} y_{p}\left(t\right) & =h_{0}\sin\left(2\pi ft\right)\\ \theta (t) & =\theta_{0}\sin\left(2\pi ft+\phi \right)=-\;\theta_{0}\cos\left(2\pi ft\right) \end{array}$$

where $h_{0}$ is the maximum heave amplitude, $\theta_{0}$ is the maximum pitch angle and *f* is the oscillation frequency. According to these assumptions and by considering sufficiently small values of the maximum pitch angle $\theta_{0}$, the lateral motion of the caudal fin $y_{c}\left(x,t\right)$ may be expressed as:

(A2) $$y_{c}\left(x,t\right)=y_{p}\left(t\right)+x\tan\left[\theta \left(t\right)\right]\approx y_{p}\left(t\right)+x\;\theta \left(t\right)$$

To look for possible similarities concerning the existence of a phase velocity also in the present case, now we compare the flapping motion under consideration with an undulatory motion. The flapping motion of the tail given by Equation (A2) may be approximated as

(A3) $$y_{c}\left(x,t\right)\approx h_{0}\sin\left(2\pi ft\right)-\theta_{0}x\cos\left(2\pi ft\right)$$

This approximated expression may be assimilated to the one for an undulatory motion of amplitude $h_{0}$ with a wavelength λ much larger than the tail length

(A4) $$y\left(x,t\right)=h_{0}\sin\left(2\pi ft-\frac{2\pi }{\lambda }x\right)\approx h_{0}\sin\left(2\pi ft\right)-\frac{2\pi }{\lambda }h_{0}x\cos\left(2\pi ft\right)$$

and, by equating the coefficients of (A3) and (A4) (i.e., $\theta_{0}=\frac{2\pi }{\lambda }h_{0}$), we may evaluate the phase velocity of the flapping motion as

(A5) $$c=f\lambda \approx 2\pi f\frac{h_{0}}{\theta_{0}}$$

In other words, for large wavelengths, the flapping tail itself may be seen as a small portion of the longer wave whose undulating motion is perceived, instantaneously, as a local oscillation given by the heave and pitch motions.

### Appendix A.2. Appendix Figures

The trailing edge trajectories for both the intact and the tailless fish are reported in Figure A1. The maximum value of the deformation velocity occurs where the alignment between the head and the rear end of the body is verified. By alignment, we intend that the rear end, i.e., the tail tip in case of intact fish, is placed along the straight line connecting the fish’s leading edge with its center of mass. The points of alignment along the trajectory, indicated by small arrows in Figure A1, are displaced from the axial direction for the tailless fish while they are exactly along the forward direction for the intact fish. For the sake of clarity, several body configurations (dashed contours) are represented to illustrate the alignment conditions. It follows the non-symmetrical configuration of the trajectory in frame (b) with respect to the symmetrical one in frame (a) of the figure. The alignment of the arrows along the locomotion axis, shows a more gentle swimming mode with respect to the tailless fish which has to implement continuously severe maneuvers to gain a sufficient performance capability and to reach a locomotion speed comparable with the one of the intact fish.

In Figure A2, we show the maximum angle of attack of the rear end for both the tailless and the intact fish together with the trace of the trailing edge trajectory. We observe a much larger angle of attack at the trailing edge for the tailless fish with respect to the intact one. Moreover, if we trace the same angles in correspondence to the head, we have a comparable behavior which confirms the need for the tailless fish to perform drastic maneuvers for a satisfactory locomotion.

For a quantitative analysis of the potential and vortical contributions to the body locomotion, we can simplify the first of the equation system (18) by moving to the r. h. s. all the terms but the one containing the unknown forward velocity *U*:

(A6) $$U\;\left(m_{11}+m_{b}\right)={P_{sh}}_{1}+{P_{v}}_{1}+V_{2}\;m_{12}+\Omega \;m_{13}=P_{1}$$

From Figure A3, we see the dominant role of the vortical contribution (red line) but also the effect of the momentum transfer from the angular to the forward direction (green line). We notice that the contribution due to the shape deformation (black line) oscillates with a zero mean value as well as the very small contribution (with nonzero mean value) of the lateral velocity (purple line).

## References

[1] Breder C. (1926). The locomotion of fishes. Zoologica.

[2] Gray J. (1933). Studies in Animal Locomotion I. J. Exp. Biol..

[3] Gray J. (1933). Studies in Animal Locomotion II. J. Exp. Biol..

[4] Gray J. (1933). Studies in Animal Locomotion III. J. Exp. Biol..

[5] Webb P.W. (1973). Effects of Partial Caudal-Fin Amputation on the Kinematics and Metabolic Rate of Underyearling Sockeye Salmon (*Oncorhynchus nerka*) at Steady Swimming Speeds. J. Exp. Biol..

[6] Plaut I. (2000). Effects of fin size on swimming performance, swimming behaviour and routine activity of zebrafish Danio rerio. J. Exp. Biol..

[7] Fu C., Cao Z.D., Fu S.J. (2013). The effects of caudal fin loss and regeneration on the swimming performance of three cyprinid fish species with different swimming capacities. J. Exp. Biol..

[8] Deakin A.G., Spencer J.W., Cossins A.R., Young I.S., Sneddon L.U. (2019). Welfare Challenges Influence the Complexity of Movement: Fractal Analysis of Behaviour in Zebrafish. Fishes.

[9] Cai L., Chen J.H., Johnson D., Tu Z., Huang Y.P. (2020). Effect of tail fin loss on swimming capability and tail beat frequency of juvenile black carp *Mylopharyngodon piceus*. Aquat. Biol..

[10] Fish F.E., Rybczynski N., Lauder G.V., Duff C.M. (2021). The Role of the Tail or Lack Thereof in the Evolution of Tetrapod Aquatic Propulsion. Integr. Comp. Biol..

[11] Audira G., Suryanto M.E., Chen K.H., Vasquez R.D., Roldan M.J., Yang C.C., Hsiao C.D., Huang J.C. (2022). Acute and Chronic Effects of Fin Amputation on Behavior Performance of Adult Zebrafish in 3D Locomotion Test Assessed with Fractal Dimension and Entropy Analyses and Their Relationship to Fin Regeneration. Biology.

[12] Lighthill J. (1960). Note on the swimming of slender fish. J. Fluid Mech..

[13] Wu T.Y. (1961). Swimming of a waving plate. J. Fluid Mech..

[14] Paniccia D., Padovani L., Graziani G., Piva R. (2023). Locomotion performance for oscillatory swimming in free mode. Bioinspir. Biomim..

[15] Reid D.A.P., Hildenbrandt H., Padding J.T., Hemelrijk C.K. (2012). Fluid dynamics of moving fish in a two-dimensional multiparticle collision dynamics model. Phys. Rev. E.

[16] Paniccia D., Graziani G., Lugni C., Piva R. (2021). The relevance of recoil and free swimming in aquatic locomotion. J. Fluids Struct..

[17] Noca F. (1997). On the Evaluation of Time-Dependent Fluid Dynamic Forces on Bluff Bodies. Ph.D. Thesis.

[18] Graziani G., Bassanini P. (2002). Unsteady Viscous Flows about Bodies: Vorticity Release and Forces. Meccanica.

[19] Wu J.Z., Ma H.Y., Zhou M.D. (2015). Vortical Flows.

[20] Paniccia D., Graziani G., Lugni C., Piva R. (2021). On the role of added mass and vorticity release for self propelled aquatic locomotion. J. Fluid Mech..

[21] Saffman P.G. (1967). The self-propulsion of a deformable body in a perfect fluid. J. Fluid Mech..

[22] Kanso E. (2009). Swimming due to transverse shape deformations. J. Fluid Mech..

[23] Eldredge J.D. (2010). A Reconciliation of Viscous and Inviscid Approaches to Computing Locomotion of Deforming Bodies. Exp. Mech..

[24] Lamb H. (1975). Hydrodynamics.

[25] Hess J.L., Smith A.M.O. (1967). Calculation of potential flow about arbitrary bodies. Prog. Aerosp. Sci..

[26] Basu B.C., Hancock G.J. (1978). The unsteady motion of a two-dimensional aerofoil in incompressible inviscid flow. J. Fluid Mech..

[27] Hess F., Videler J.J. (1984). Fast continuous swimming of Saithe (*Pollachius virens*): A dynamical analysis of bending moments and muscle power. J. Exp. Biol..

[28] Videler J., Hess F. (1984). Fast continuous swimming of two pelagic predators saithe (*Pollachius virens*) and mackerel (*Scomber scombrius*): A kinematic analysis. J. Exp. Biol..

[29] Maertens A., Triantafyllou M., Yue D. (2015). Efficiency of fish propulsion. Bioinspir. Biomim..

[30] Jurczyk K., Piskur P., Szymak P. (2020). Parameters identification of the flexible fin kinematics model using vision and genetic algorithms. Pol. Marit. Res..

[31] Li N., Liu X., Su Y. (2017). Numerical study on the hydrodynamics of thunniform bio-inspired swimming under self-propulsion. PLoS ONE.

[32] Lighthill J. (1969). Hydromechanics of aquatic animal propulsion: A survey. Annu. Rev. Fluid Mech..

[33] Wu T. (2011). Fish Swimming and Bird/Insect Flight. Annu. Rev. Fluid Mech..

[34] Smits A. (2019). Undulatory and oscillatory swimming. J. Fluid Mech..

[35] Akoz E., Moored K.W. (2018). Unsteady propulsion by an intermittent swimming gait. J. Fluid Mech..

[36] Floryan D., Buren T.V., Rowley C.W., Smits A.J. (2017). Scaling the propulsive performance of heaving and pitching foils. J. Fluid Mech..

[37] Xiong G., Lauder G.V. (2014). Center of mass motion in swimming fish: Effects of speed and locomotor mode during undulatory propulsion. Zoology.

[38] Lauder G.V. (2015). Fish Locomotion: Recent Advances and New Directions. Annu. Rev. Mar. Sci..

[39] Paniccia D., Padovani L., Graziani G., Piva R. (2021). The performance of a flapping foil for a self-propelled fishlike body. Sci. Rep..

[40] Bale R., Hao M., Bhalla A.P.S., Patel N., Patankar N.A. (2014). Gray’s paradox A fluid mechanical perspective. Sci. Rep..

[41] Zhang J. (2017). Footprints of a flapping wing. J. Fluid Mech..

[42] Ahlborn B., Harper D., Blake R., Ahlborn D., Cam M. (1991). Fish without Footprints. J. Theor. Biol..

[43] Paniccia D., Graziani G., Lugni C., Piva R. (2022). The fish ability to accelerate and suddenly turn in fast maneuvers. Sci. Rep..

[44] Webb P.W. (1971). The swimming energetics of trout. I. Thrust and power output at cruising speeds. J. Exp. Biol..

[45] Chopra M.G. (1976). Large amplitude lunate-tail theory of fish locomotion. J. Fluid Mech..

[46] Webb P.W. (1992). Is the High Cost of Body/Caudal Fin Undulatory Swimming due to increased Friction Drag or Inertial Recoil?. J. Exp. Biol..

[47] Webb P., Weihs D. (2015). Stability versus Maneuvering: Challenges for Stability during Swimming by Fishes. Integr. Comp. Biol..

[48] Borazjani I. (2013). The functional role of caudal and anal/dorsal fins during the C-start of a bluegill sunfish. J. Exp. Biol..

[49] Bhalla A.P.S., Bale R., Griffith B.E., Patankar N.A. (2013). A unified mathematical framework and an adaptive numerical method for fluid-structure interaction with rigid deforming and elastic bodies. J. Comp. Phys..

